# Semi-Supervised Graph Attention Network for Screw Pump Fault Diagnosis: Revealing the Dynamic Coupling of Multi-Source Information

**DOI:** 10.3390/e28030338

**Published:** 2026-03-18

**Authors:** Weigang Wen, Jingqi Qin, Qiuying Chang

**Affiliations:** School of Mechanical, Electronic and Control Engineering, Beijing Jiaotong University, Beijing 100044, China; wgwen@bjtu.edu.cn (W.W.); qychang@bjtu.edu.cn (Q.C.)

**Keywords:** semi-supervised learning, graph attention networks, multi-source information, screw pump, fault diagnosis

## Abstract

The screw pump is a critical device for elevating downhole petroleum to the surface, and screw pump failure can significantly disrupt the production of oil wells. Due to the complex structure of the screw pump, the same pump fault can cause different changes in the monitoring parameters, and different faults can also cause the same parameter change. In consequence of the complexity, it requires a large amount of labeled data for a diagnosis model to achieve fault diagnosis of a screw pump in practical application. Aiming for this kind of condition, we discovered the dynamic coupling effect between multi-source information through detailed research on the collected data of screw pumps. To fully leverage the information dynamic coupling (IDC) effect, a semi-supervised learning graph attention network (SSL-GAT) fault diagnosis method is proposed. This approach integrates the semi-supervised learning framework and graph attention neural network for the fault diagnosis of a screw pump. The experimental validation of the SSL-GAT method demonstrates its outstanding performance in screw pump fault diagnosis.

## 1. Introduction

A screw pump is widely used to extract oil in oil wells. It plays an important role in the steady output of an oilfield. In oil production and the distribution process, screw pump failures occur frequently, seriously affecting oil production Such failures not only significantly compromise system operational efficiency and economic performance, but also pose serious substantial safety hazards. The evolution of screw pump fault diagnosis technology has progressed from traditional signal processing to data-driven intelligent approaches. Early diagnostics primarily relied on manual inspections and basic signal analysis. In recent years, however, significant advancements have been made in this field driven by the advancement of Industry 4.0 and artificial intelligence, emphasizing real-time monitoring, interpretability, and robustness.

The initial diagnostic techniques for screw pumps focused on the physical analysis of mechanical failures, such as shaft fractures, bearing wear, and pump body leaks. Methods included vibration signal monitoring and spectral analysis. During this period, fault diagnosis relied heavily on empirical knowledge, resulting in low accuracy and significant susceptibility to noise interference [1]. Yang [2] analyzed material fatigue, stress distribution, and wear patterns by dissecting failed pump bodies. Typical tools include scanning electron microscopy (SEM) and stress simulation software, with a focus on fracture mechanisms in the shafts and bearings. This approach is suitable for post-failure diagnosis but cannot provide real-time early warnings. A research study [3] introduced wavelet analysis or Fourier transform for processing vibration data, though its application remained limited, primarily used for generic hydraulic pump fault detection. Machine learning had not yet become widespread during this period, with diagnostics largely relying on human expertise. Shawki [4] analyzed early failures in high-pressure screw pumps, concluding that shaft fatigue fractures stemmed from misalignment. Through comprehensive analysis (including finite element simulation), he proposed alignment correction methods. Jim [5] evaluated the health status of screw pumps based on dynamic wellbore characteristics, process parameters, and well testing data. Cui Jinbang [6] adopted a “Working Condition Testing–Replay Diagnostics” methodology to investigate screw pump operational states. Wang Ruihua [7] achieved screw pump condition diagnosis through electrical parameter analysis and established a method for identifying leakage sources. Zhang Peng [8] developed an intelligent monitoring system for oil extraction screw pumps, achieving real-time monitoring of pump operational status by collecting and analyzing key data such as pump pressure, electrical parameters, and liquid production rates.

With the introduction of machine learning methods, diagnostics have shifted from qualitative post-event analysis to a semi-automated, data-driven model. This approach focuses on real-time identification of screw pump failures under complex oilfield conditions such as high sand content, high wax content, and heavy oil. Common failures remain primarily shaft breakage, wear, cavitation, and leakage. Diagnostics now incorporate quantitative feature extraction and pattern recognition, achieving higher accuracy compared to traditional methods. A 2020 study [9] proposed a random forest-based diagnostic method achieving 92.86% accuracy by analyzing sensor data (e.g., vibration, pressure), suitable for real-time fault detection in oil well production. Concurrently, fuzzy logic and neural networks began application—such as algorithms for pumping unit fault diagnosis—extending to similar screw pump systems. This phase emphasized data storage and processing, such as the Hadoop-based HDFS system for large-scale vibration data analysis. Zhang [10] discovered that fuzzy logic could handle uncertain and ambiguous information, providing an effective means for addressing complex failures in pump units. Neural networks, leveraging robust self-learning and pattern recognition capabilities, provided high-precision solutions for fault classification and identification in pumping units. Dong [11] proposed a fault diagnosis method for submersible screw pumps based on probabilistic neural networks (PNN) and developed a corresponding diagnostic system. A dataset of 142 fault cases was collected to train the model and validate its accuracy, with test results demonstrating a diagnostic accuracy rate of 90.5%.

Benefiting from the explosive application of deep learning (DL) and large-scale industrial field data collection, screw pump fault diagnosis has shifted toward end-to-end deep feature learning, multimodal fusion, data imbalance handling, and cross-domain adaptation, significantly enhancing accuracy and robustness. In a 2024 study [12], a model-driven approach constructed a fault data model (FDM) to achieve precise screw pump diagnostics through multi-source feature extraction (e.g., vibration, current, pressure). Luo E [13] proposed a multi-scale CNN diagnostic method for precision screw pumps. By collecting triaxial vibration signals and constructing an MSCNN model combined with signal processing, it achieved 92% accuracy—significantly outperforming traditional BP/CNN approaches—and was applied to improve stability in new energy vehicle stator coating production lines. Li X [14] proposed an ESPCP operational condition diagnosis framework using CNN-ResNet-RF. With 360 sets of field records, TimeGAN generated minority class samples. CNN-ResNet extracted deep features combined with RF classification, achieving 97.3% validation accuracy and 98.5% Macro-F1 score, substantially reducing misclassifications of leaks and low parameters. Field verification showed 100% consistency with maintenance records.

Drilling pump failures primarily occur in the hydraulic end, with common types including valve disc leakage, cylinder liner and piston wear, vane leakage, pump valve sticking, bearing failure, cavitation, and crosshead wear. Tang [15] proposed a drilling pump fluid-end fault diagnosis method based on generalized S-transform (GST) and convolutional neural networks (CNN). By introducing batch normalization and optimizing the number of fully connected layer neurons, this approach analyzes the identification performance for normal, minor damage, and severe damage states of drilling pump fluid ends. Guo [16] proposed WaveletKernelNet-Convolutional Block Attention Module-BiLSTM for intelligent fault diagnosis of drilling pumps. The convolutional block attention module embedded in WaveletKernelNet-CBAM adjusts weights to enhance feature representations in both channel and spatial dimensions. Li [17] addressed the challenge of extracting fault features from drilling pump fluid-end timing signals under complex operating conditions by proposing a method to generate images representing multidimensional timing signals. Guo J [18] introduced a novel parallel deep neural network for drilling pump fault diagnosis. It integrates the convolutional block attention module with AlexNet and synchronizes with the anomaly transformer model to meticulously explore both the time domain and time-frequency domain of signals. Dai [19] proposed a fault diagnosis method based on time–frequency analysis and convolutional neural networks. The continuous wavelet transform (CWT) was employed to convert collected vibration signals into time–frequency maps. A fault diagnosis model based on SqueezeNet was developed to identify faults, effectively achieving precise fault diagnosis.

Heat pumps serve as core equipment for achieving building decarbonization and efficient energy utilization. Their fault diagnosis technology has evolved from traditional manual inspections to data-driven intelligent systems, demonstrating high maturity and rapid advancement toward intelligence. Boahen S [20] investigated refrigerant charge faults and their impact on variable-speed heat pumps, identifying the most accurate method for detecting refrigerant charge faults using compressor discharge temperature, outdoor inlet water temperature, and compressor speed as inputs, and refrigerant charge as the output, by comparing multiple linear regression and multilayer perceptron models. Borges [21] introduced and evaluated a scalable evaporator fouling method for air-to-water heat pumps. To utilize artificial neural networks for detecting evaporator fouling during operation, a transient model of the refrigerant cycle must be provided as training data. Llopis-Mengual B [22] proposed a method using virtual sensors to detect evaporator fouling in air-to-water heat pumps. The virtual sensor was calibrated using simulated fault-free performance profiles and tested on heat pumps equipped with variable-speed components. The method’s effectiveness was evaluated through simulations and induced evaporator fouling experiments.

Screw pump fault diagnosis techniques share similarities with drilling pumps and heat pumps, such as vibration signal analysis and AI applications. However, due to differing application scenarios, significant variations exist. Drilling pump failures primarily involve vibration-related issues (e.g., shoe wear, pump valve leakage), mud loss under complex conditions, and drill cuttings problems. Heat pump diagnostics primarily focus on soft faults (e.g., refrigerant leaks, heat exchanger scaling, compressor valve plate damage) and electrical faults (e.g., reverse phase protection). Screw pump failures are mainly mechanical (e.g., shaft breakage, screw wear, bearing failure), cavitation, and leakage. They are highly influenced by viscosity. Screw pump signals are diverse, including vibration, current, pressure, load, and speed. Their fault diagnosis involves multi-source fusion technology.

Multi-source information fusion (MSIF) is a data processing technique designed for integrating data from multiple homogeneous or heterogeneous sources, and its application in fault diagnosis has become increasingly prevalent [23,24,25]. Yu [26] proposed an end-to-end machine fault diagnosis framework that employs a cross-attention mechanism for multi-source information fusion and utilizes a weighted activation mechanism for fault identification. Gong [27] leveraged a hierarchical vision transformer based on multi-source information fusion to extract fault features from time–frequency representations, thereby enabling effective fault diagnosis. Zhang [28] applied a residual pyramid algorithm for multi-source data fusion, resulting in a novel multi-source fusion-based fault diagnostic methodology. Due to the structural complexity of mechanical systems and the diversity of information acquisition, a single information processing method is no longer sufficient. The information collected at actual engineering sites is highly dimensional. Compared to fault diagnosis based on single-source information, mechanical equipment fault diagnosis methods utilizing multi-source information fusion offer higher accuracy and greater stability.

Graph neural networks (GNNs) exhibit significant advantages in processing non-Euclidean data, offering a unified representation framework for multi-source data integration [29,30]. Yao J [31] proposed a novel fault diagnosis approach for rolling bearings utilizing fast temporal graph convolutional networks (FTGCNs), introducing graph convolutional layers with rapid kernels to achieve efficient fault classification. Kavianpour M [32] developed a semi-supervised fault diagnosis method based on ARMA graph convolution, adversarial adaptation, and multi-layer multi-kernel local maximum mean discrepancy (MK-LMMD). Jiang L [33] introduced the multi-head graph attention network (MHGAT) to enhance and fuse node features for bearing fault diagnosis. Cao S [34] presented the spiking graph attention network (Spiking-GAT) for intelligent fault diagnosis of planetary gearboxes. Liu L [35] proposed a graph dynamic autoencoder (GDAE) for fault diagnosis applications. Feng Y [36] utilized a full-graph autoencoder to transform multivariate time series into graph data that incorporates prior knowledge, enabling real-time system condition monitoring. Nima Rezazadeh [37] introduced GAT-CAMDA, a novel framework for the structural health monitoring (SHM) of composite materials under temperature-induced variability, leveraging the powerful feature extraction capabilities of graph attention networks (GATs) and advanced domain adaptation (DA) techniques. By combining maximum mean discrepancy (MMD) and correlation alignment (CORAL) losses with a domain-discriminative adversarial model, the framework achieves scalable alignment of feature distributions across temperature domains, ensuring robust damage detection. 

However, during screw pump operation, variations in one parameter induce corresponding changes in others, and the coupling effects among different information sources could vary depending on the specific fault condition of the screw pump. In other words, the interrelationships among multi-source information are dynamic and modulated by the fault state of the screw pump. Based on this principle, this study proposes a screw pump fault diagnosis approach grounded in the information dynamic coupling (IDC) effect, utilizing graph attention networks (GAT) to reveal dynamic coupling relationships among information sources, thereby implementing intelligent fault diagnosis in the practical industrial field. Based on this, this study further proposes a semi-supervised learning graph attention network (SSL-GAT) method that integrates multi-source information for screw pump fault diagnosis. The main content of this paper is organized as follows: Section 1 presents the introduction; Section 2 details the SSL-GAT-based screw pump fault diagnosis framework; Section 3 provides experimental validation; and Section 4 concludes the study.

## 2. Semi-Supervised Learning Graph Attention Networks (SSL-GAT) Framework for Screw Pump Fault Diagnosis

**Figure 1:**
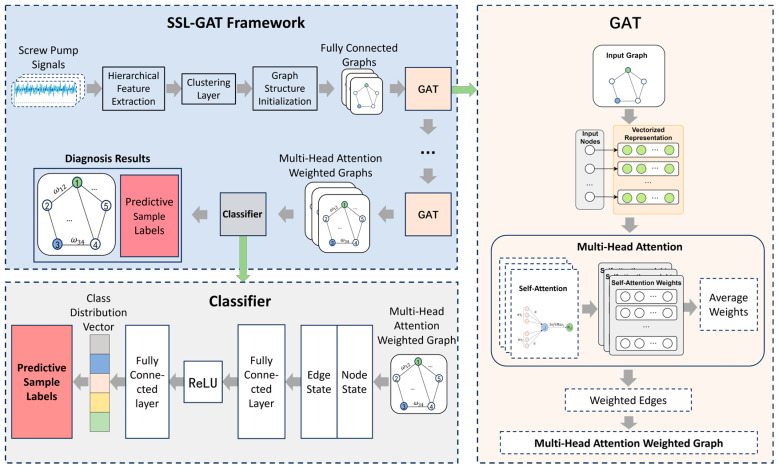
Semi-supervised learning graph attention network (SSL-GAT) framework flow.

This paper addresses the challenge of screw pump fault diagnosis under limited labeled samples by leveraging multi-source information. A semi-supervised learning framework integrated with graph attention networks (SSL-GAT) is proposed, as illustrated in Figure 1.

Semi-supervised learning (SSL) integrates a small set of labeled samples alongside a large volume of unlabeled data to train diagnostic models. The SSL process typically involves four iterative stages: initially, a base model is trained using the limited labeled dataset; subsequently, this trained model is employed to predict labels for the abundant unlabeled samples, wherein high-confidence predictions are assigned pseudo-labels; then these pseudo-labeled samples are incorporated into the original training set to form an expanded dataset, which is used to retrain the model; and finally the model is iteratively updated until its performance converges or a predefined iteration limit is reached. By cyclically utilizing unlabeled data, this approach enhances model performance and significantly reduces reliance on labeled data, thereby improving the cost-effectiveness of training graph neural network algorithms for screw pump fault diagnosis.

In this study, labeled data constitutes the smaller dataset *L*, which is partitioned into training, validation, and test sets; unlabeled data constitutes the larger dataset *U*, which is similarly partitioned. A base model f is randomly initialized. The confidence threshold τ is set to 0.95, the maximum iteration count T to 10, the maximum addition ratio per round α to 0.1, and the threshold decay factor e to 0.98. Each iteration involves training a model $f_{t}$ on the current full labeled dataset *L*. A forward prediction is then performed for each sample x in the remaining unlabeled dataset *U*, producing a class probability distribution p(y∣x). A pseudo-label $y^{\prime }=arg\;{max}_{y}p(y|x)$ is generated, and its confidence $c={max}_{y}p(y|x)$ is calculated. A pseudo-label is deemed reliable only if its confidence meets or exceeds the current threshold τ (c ≥ τ). All samples satisfying this criterion are collected to form a candidate pseudo-label set *S*. Should the size of *S*, |*S*|, exceed α×|*U*|, the samples are sorted in descending order of confidence c, and only the top α × |*U*| highest-confidence samples are retained, thereby regulating the number of additions. Samples (x, y’) from *S* are permanently incorporated into the labeled dataset *L* and concurrently removed from the unlabeled dataset *U*. Upon completion of all iterations, the final model, $f_{T}$, is the output.

The graph neural network model for screw pump fault diagnosis utilizes graph-structured data as the information carrier, where both nodes and edges are capable of storing and transmitting information, thereby aligning closely with the objective of multi-dimensional information fusion in screw pumps. A graph is constructed based on multi-source information samples from the screw pump, with each dimension of information represented as a node and various statistical metrics serving as node features. The relationships between information, induced by the “information dynamic coupling” (IDC) effect, are modeled as edges. An attention mechanism is employed to compute attention scores between nodes, thereby constructing a graph attention network that quantitatively characterizes the IDC effect.

### 2.1. Graph Initialization of the Multi-Source Information Graph Attention Network for Screw Pump

**Figure 2:**
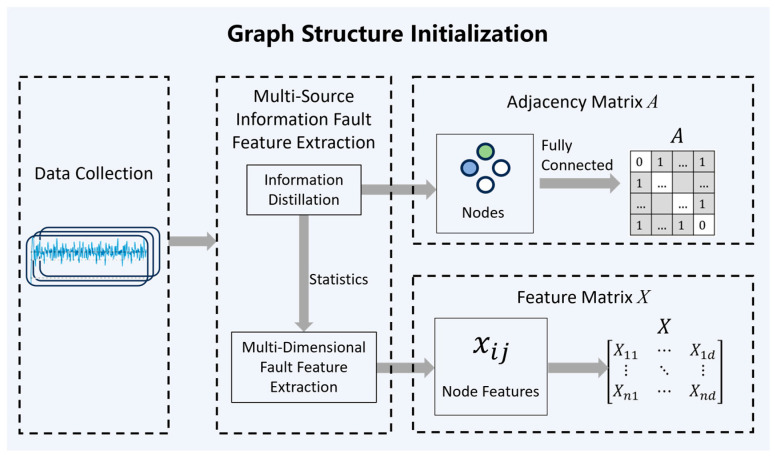
Graph structure initialization flow.

The multi-source information collected from the actual engineering site of the screw pump is transformed into a graph, followed by graph structure initialization, as illustrated in Figure 2. Initially, data acquisition captures four types of information: current, rotational speed, load, and oil pressure. After information distillation, each dimension of data is represented as a node in the graph. For each node, three statistical metrics—mean, variance, and peak-to-peak value—are computed as node features, resulting in the formation of the data matrix M and the node feature matrix X, thereby completing node initialization. Assuming the existence of potential edges between all nodes, a fully connected graph G is constructed, and the adjacency matrix A is generated by setting all off-diagonal elements to 1, thus achieving edge initialization.

### 2.2. Graph Construction of the Multi-Source Information Graph Attention Network for the Screw Pump

**Figure 3:**
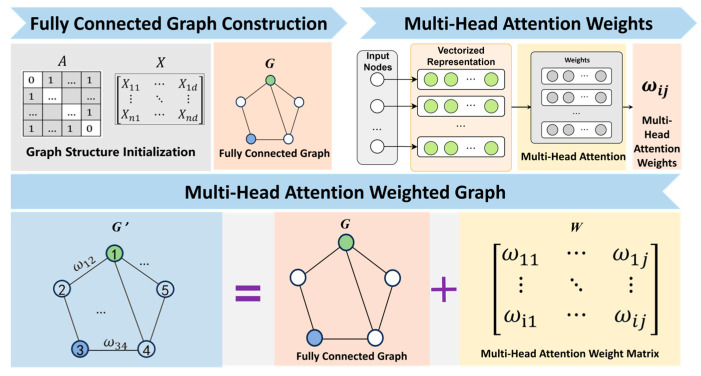
Multi-head attention weighted graph construction flow.

To evaluate and leverage the intrinsic features of each dimension of screw pump information and the dynamic coupling among multi-source information, a graph attention network for multi-source screw pump data is constructed, as illustrated in Figure 3. The process begins with graph structure initialization, where the adjacency matrix A and feature matrix X are established to form an undirected fully connected graph G. Subsequently, a multi-head attention mechanism is employed to compute the multi-head attention weight matrix W for graph G. Finally, the undirected weighted graph G is integrated with the multi-head attention weight matrix W to generate the multi-head attention weighted graph (MHAWG) G’.

### 2.3. Calculation of Multi-Source Information Dynamic Coupling Relationships for the Screw Pump Based on the Graph Multi-Head Attention Mechanism

**Figure 4:**
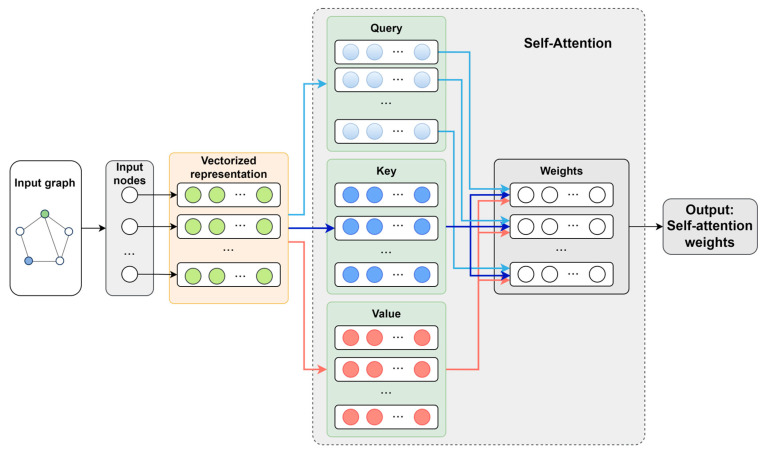
Attention mechanism structure.

Utilizing multi-dimensional data collected from the screw pump—including current, rotational speed, load, and oil pressure—each dimension is represented as a node, with statistical metrics serving as node features. The initial input graph *G(V*, *E)* for the screw pump’s multi-source information is constructed through graph network initialization. Employing an attention mechanism, the attention relationship between each node vi and its neighboring nodes vj within the set N(i)  is computed, thereby establishing a graph attention network, as illustrated in Figure 4. This approach enables the adaptive determination of edge weights between nodes, allowing for the dynamic learning of the associative characteristics between neighboring nodes and the central node. The graph attention mechanism assigns a learned weight coefficient to each edge, referred to as the graph attention coefficient $e_{ij}$:

(1) $$e_{ij}=a\left(Wh_{i},Wh_{j}\right),j\in N_{i}$$

where *W* denotes the shared learnable weight matrix, *h_i_* and *h_j_* represent the node features, and a( ) is the attention mechanism. The graph attention coefficients are subsequently normalized using the Softmax function to yield the final weights:

(2) $$\alpha_{\left(ij\right)}={softmax}_{j}\left(e_{ij}\right)=\frac{\exp\left(e_{ij}\right)}{\sum_{k\in N\left(i\right)}\exp\left(e_{ik}\right)}$$

To enhance the diversity of the graph attention mechanisms, multiple independent self-attention modules are applied to distinct node feature spaces, enabling each edge to acquire representations from different attention subspaces. Ultimately, this approach constructs a multi-head attention weight matrix corresponding to the original edges, which can be expressed as follows:

(3) $$h_{i}^{\prime }=\sigma (\frac{1}{K}\sum_{k=1}^{K}\sum_{j\in N\left(i\right)}\alpha_{k(ij)})$$

where σ denotes the activation function, which can be implemented as either the ELU or ReLU function. Specifically, the features extracted by each attention head are aggregated via mean pooling to obtain the embedding representations of all nodes and edges within each layer of the graph. The attention weight matrix quantifies the influence of neighboring nodes on the source node, thereby facilitating the assessment of dynamic coupling relationships among various dimensions of screw pump information.

Consequently, the multi-source data collected from the screw pump during each time interval forms a graph *G(V*, *E)*. Through semi-supervised learning (SSL), the shared learnable weight matrix W is optimized, resulting in a multi-layer graph embedding based on a graph attention network—namely, the multi-head attention weighted graph (MHAWG). This representation can be utilized for graph classification, enabling the integration of multi-source information for screw pump fault identification and diagnosis under limited labeled sample conditions using a graph attention network.

## 3. Experiments and Results

### 3.1. Data Description

The raw data for the screw pump are provided by a petroleum machinery company. The data originate from oil wells numbered “A12_3082900” to “Z70_3060621” in an oilfield of China, encompassing multidimensional information such as voltage, current, torque, load, and rotational speed under various operating conditions, comprising both labeled and unlabeled datasets. After data preprocessing, four parameters—current, rotational speed, load, and oil pressure—were selected as graph network nodes. For each parameter, three statistical features—mean, variance, and peak-to-peak value—were extracted as node attributes. Ten common types of screw pump faults encountered in site were identified, as detailed in Table 1.

### 3.2. Labeled Samples Sensitivity Analysis

**Figure 5:**
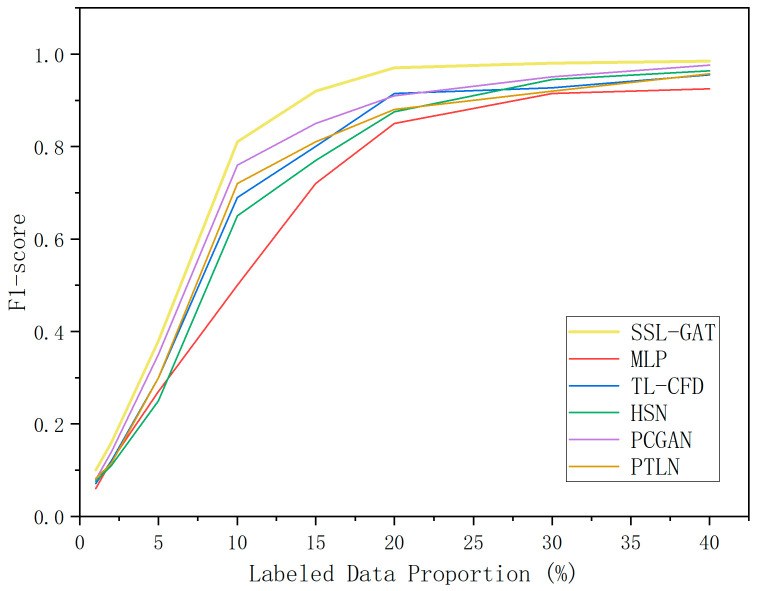
The F1 score of the proposed method and the comparison method under different proportion of labeled data.

To assess the impact of varying proportions of labeled samples on the performance of semi-supervised learning (SSL) fault diagnosis models, this study compared the proposed SSL-GAT approach with multilayer perceptron (MLP) and another four most recently proposed (2025–2026) fault diagnosis classification algorithms: transfer learning-based concurrent fault diagnosis (TL-CFD) [38], hierarchical stochastic network (HSN) [39], physically constrained generative adversarial network (PCGAN) [40], progressive transfer learning network (PTLN) [41]. Each of these five methods was implemented within a semi-supervised learning framework, with the proportion of labeled data set at 1%, 2%, 5%, 10%, 15%, 20%, 30%, and 40% of the total dataset. Under identical experimental conditions, this study conducted fault diagnosis experiments on screw pumps. The F1-scores and corresponding F1-score curves for all six fault diagnosis methods at different labeled data ratios are presented in Table 2 and Figure 5.

The experimental results indicate the following: (1) among the six comparative methods, SSL-GAT consistently achieves the highest F1-score across varying proportions of labeled data, reaching a peak value of 0.977, thereby demonstrating superior performance; (2) when the proportion of labeled data falls below 5%, all six methods yield F1-scores below 0.4, suggesting that the semi-supervised learning models fail to train effectively and cannot accurately diagnose faults under these conditions. For SSL-GAT, the F1-score increases significantly as the proportion of labeled data rises from 10% to 20%. Once the labeled data proportion exceeds 20%, the F1-score curve reaches an inflection point, and the marginal benefit of additional labeled data diminishes markedly, indicating that the model performance approaches its theoretical upper limit with minimal room for further improvement. The remaining four methods exhibit a similar trend, with the inflection point occurring when the labeled data proportion ranges from 10% to 30%.

These findings demonstrate that the SSL-GAT method offers robust fault diagnosis capabilities and achieves an optimal balance between model performance enhancement and annotation cost when the proportion of labeled data is relatively low, around 20%.

### 3.3. Efficacy Validation of SSL-GAT for Sparse Labeled Samples

To evaluate the performance of the proposed semi-supervised learning graph attention network (SSL-GAT) approach, SSL-GAT was compared with five common fault diagnosis classification algorithms: HSN, TL-CFD, PCGAN, PTLN, and MLP. All methods were assessed under identical conditions: all labeled data were evenly split into two subsets, designated as the training set and the test set for the model. Based on the experimental results presented in Section 3.1, the training set was further partitioned into 20% labeled data and 80% unlabeled data.

**Figure 6:**
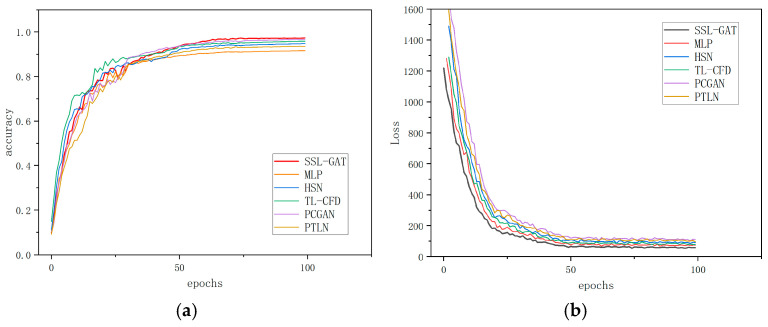
Results of different fault diagnosis methods: (**a**) accuracy; (**b**) loss.

The test accuracy and loss convergence curves during the training process for these six fault diagnosis methods are presented in Figure 6. Since SSL-GAT is capable of leveraging the entire training dataset, including both labeled and unlabeled samples, the results indicate that among the six approaches, SSL-GAT achieved an accuracy exceeding 97.5% and a loss below 65, both representing the best performance. These findings demonstrate that the SSL-GAT method exhibits superior capability for screw pump fault diagnosis.

**Figure 7:**
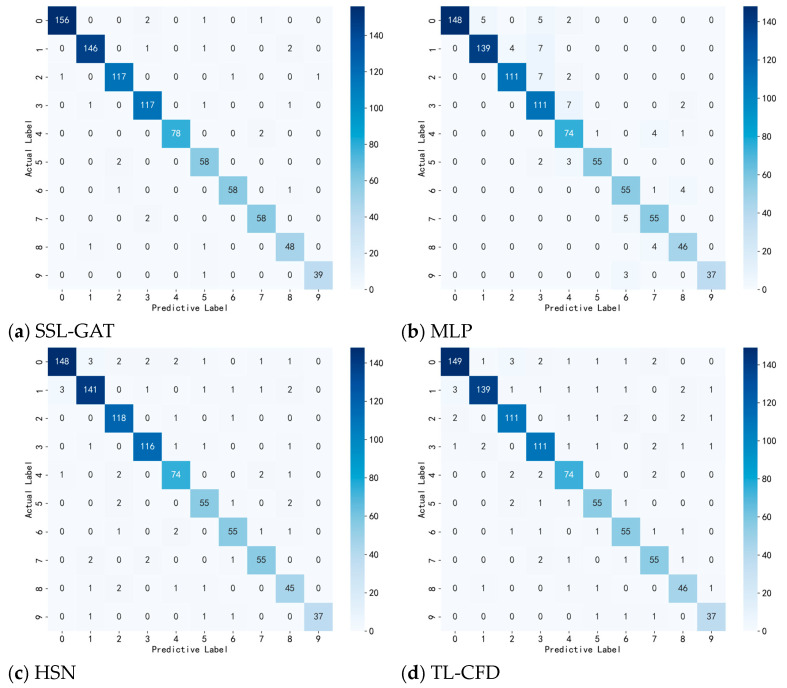

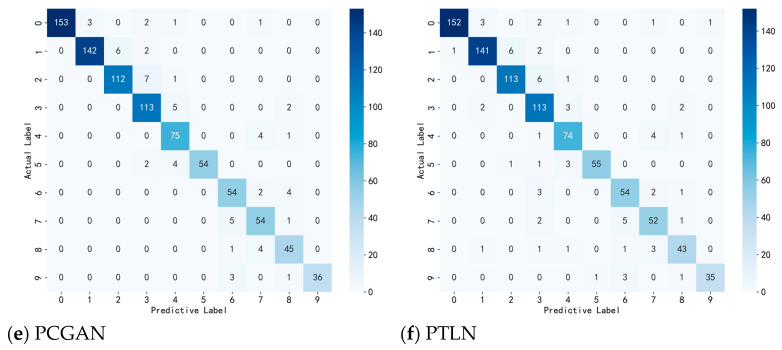
Confusion matrices of different methods.

Confusion matrix—also known as the error matrix—is a widely used representation to evaluate the performance of classification models. For an n-class classification problem, this matrix is presented in the form of an n×n table. Figure 7 illustrates the confusion matrices for SSL-GAT and other fault diagnosis methods under the experimental condition where the training set comprises 80% of the data. It is evident that the SSL-GAT confusion matrix exhibits the highest number of true positive predictions, indicating that this method demonstrates superior diagnostic performance.

**Figure 8:**
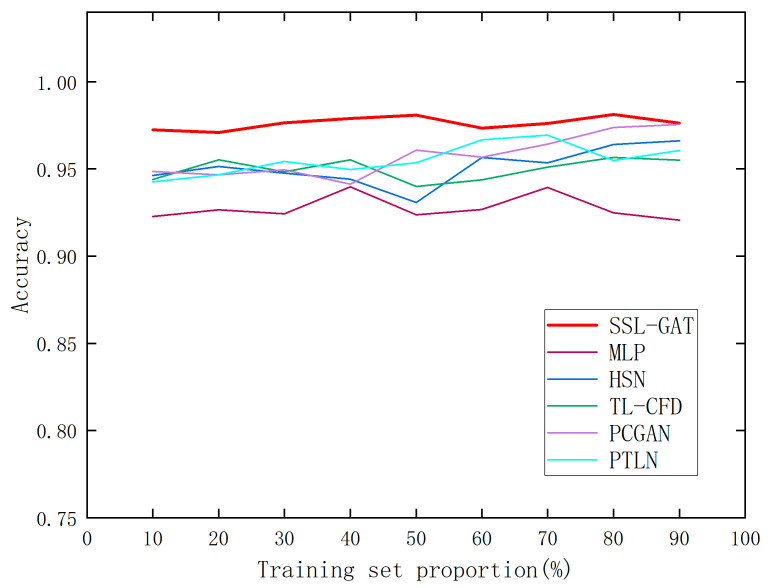
Accuracy of the SSL-GAT and comparison methods with different training set ratios.

The proportion of the training set in the experimental datasets was systematically reduced from 90% to 10% in decrements of 10%, while the proportion of the test set was correspondingly increased from 10% to 90% in increments of 10%. The fault diagnosis accuracies of various diagnostic methods under each training and test set split are presented in Table 3, with the corresponding accuracy trends illustrated in Figure 8.

**Figure 9:**
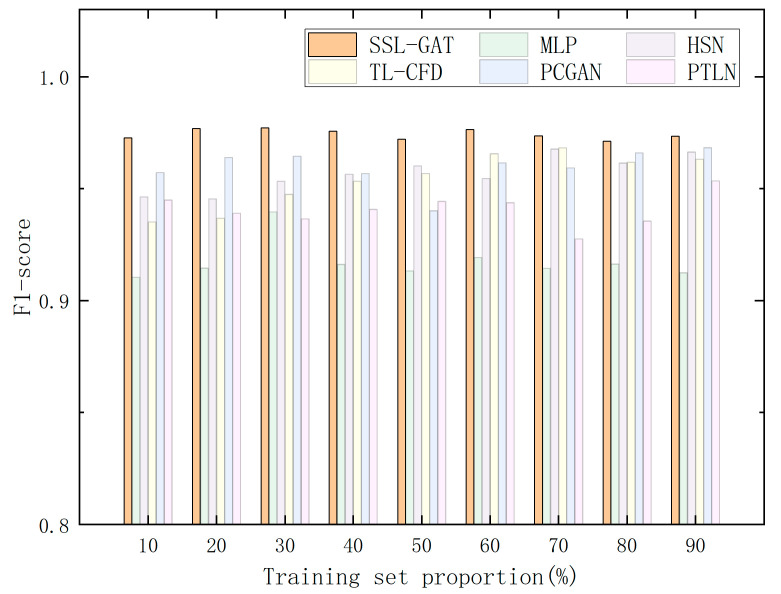
The F1-scores of the proposed method and the comparison method at different training set shares.

To evaluate the performance of the SSL-GAT model, the F1-score is employed as an evaluation metric, which is derived from the harmonic mean of precision (P) and recall (R). The F1-score ranges from 0 to 1, with higher values indicating a more optimal balance between precision and recall, thus reflecting superior overall model performance. An F1-score below 1 suggests potential for further improvement. Under the specified proportions of training and testing datasets, the F1-scores for various fault diagnosis methods are presented in Table 4. The variation in F1-scores is illustrated in Figure 9.

A comparative analysis of these six diagnosis methods demonstrates that the SSL-GAT approach consistently outperforms alternative techniques across multiple evaluation metrics, including training accuracy and loss, confusion matrix results, accuracy at varying training set proportions, and F1-score. Consequently, SSL-GAT exhibits superior performance in screw pump fault diagnosis applications.

To investigate the impact of the number of heads in multi-head attention on model performance, this study trained models with varying head counts set to 4, 6, 8, 10, and 12. When the number of heads was low, increasing it improved model performance. However, this improvement was not linear but exhibited diminishing marginal returns. Beyond eight heads, further increases in head count yield negligible improvements in model performance. Consequently, this study selected eight heads for model training.

### 3.4. Visualization Analysis

**Figure 10:**
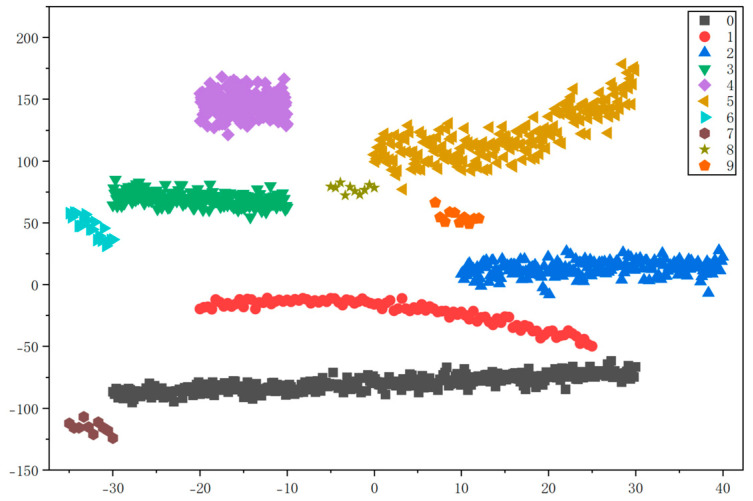
Clustering results of the t-SNE visualization of MHAWG.

**Figure 11:**
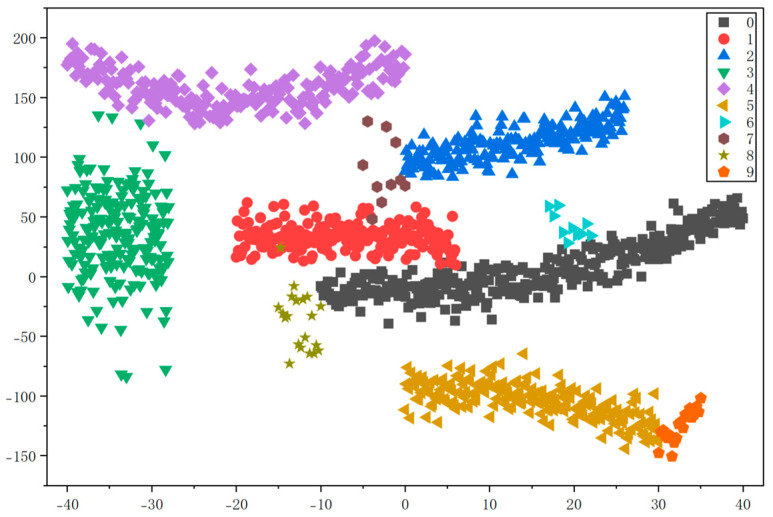
Clustering results of t-SNE visualization of KNNG.

To evaluate the accuracy of the multi-head attention weighted graph (MHAWG) constructed in chapter 2.1 and the K-nearest neighbor graph (KNNG) for screw pump fault diagnosis, comparative experiments were conducted using a support vector machine with the kernel parameter set to ‘rbf’. The clustering performance of the two graph construction methods was assessed through visualization with t-SNE, as illustrated in Figure 10 and Figure 11.

As shown in Figure 10, although a small number of outlier points are present in the clusters generated by MHAWG, the ten fault types are distinctly and independently separated. In contrast, Figure 11 demonstrates that the graph constructed using the KNNG method exhibits a certain degree of data overlap during clustering. These results indicate that the MHAWG method achieves superior clustering performance for fault diagnosis

The Calinski–Harabasz Index (CHI) was employed to evaluate the quality of clustering results. This is a statistical measure used to assess the quality of clustering results, particularly useful when determining the optimal number of clusters. The CHI is based on the ratio of intra-cluster dispersion to inter-cluster dispersion. A higher CHI value indicates a more distinct clustering structure, characterized by greater differences between clusters and smaller differences within clusters. Therefore, a good clustering result should exhibit a high CHI. The formula for calculating the CHI is as follows:

$$CHI(k)=\frac{B_{k}/(k-1)}{W_{k}/(n-k)}$$

where $B_{k}$ is cluster dispersion, which is the weighted sum of the dispersion between each cluster centroid and the overall centroid. A higher value indicates greater differences between clusters. $W_{k}$ is intra-cluster dispersion, defined as the weighted sum of the dispersion of all points within each cluster relative to its centroid. A smaller value indicates greater cohesion within the cluster. k is the number of clusters. n is the total number of samples.

MHAWG achieved its maximum CHI value of 0.91 at 10 clusters, while KNNG reached its maximum CHI value of 0.80 at 10 clusters. This indicates that MHAWG is more effective for fault diagnosis, thereby validating its superiority.

## 4. Conclusions

For the purpose of the few labeled data samples training fault diagnosis model in an engineering application, this paper addresses the challenge of fault diagnosis of oilfield screw pumps by leveraging the information dynamic coupling (IDC) effect and the multi-source information fusion capability of the graph attention network (GAT). Then, a semi-supervised learning graph attention network (SSL-GAT) framework is proposed to enable multi-source information fusion for screw pump fault diagnosis. The main findings are as follows:

(1) Graph-structure data demonstrates superior performance in representing complex associations, handling heterogeneous information, and supporting intelligent decision-making, thus providing an effective approach for multi-source information representation.

(2) The graph attention network quantifies the information dynamic coupling (IDC) effect by computing weight matrices across different information dimensions, enabling effective multi-source information fusion from both intra- dimensional and inter-dimensional perspectives.

(3) The SSL-GAT screw pump fault diagnosis framework achieves reliable fault identification under conditions of few labeled samples, indicating that this method warrants further research and broader application.

## Figures and Tables

**Table 1:** Fault types of screw pump.

Fault Identification Number	Fault Classification	Root Cause Analysis
0	Oil rod breakage	Excessive torque/tension
1	Oil pipe leakage	Oil pipe corrosion
2	Oil pipe breakage	Anti-rotation anchor damage
3	Oil pipe waxing	Oil well contains high wax
4	Stator swelling	Absorption, heating, expansion
5	Stator delaminating	Low bonding strength
6	Pump leakage	Stator wear and aging
7	Pump blockage	Excessive over-fitment
8	High parameters	Excessive fluid supply capacity
9	Low parameters	Insufficient fluid supply capacity

**Table 2:** The F1 score of the proposed method and the comparison method under different proportion of labeled data.

Labeled Data Proportion (%)	SSL-GAT	MLP	HSN	TL-CFD	PCGAN	PTLN
1	0.105	0.06	0.071	0.076	0.08	0.082
2	0.161	0.12	0.12	0.11	0.14	0.115
5	0.382	0.27	0.303	0.25	0.35	0.312
10	0.813	0.501	0.69	0.65	0.76	0.72
15	0.925	0.72	0.809	0.77	0.85	0.81
20	0.971	0.85	0.915	0.875	0.91	0.88
30	0.975	0.915	0.927	0.945	0.951	0.92
40	0.977	0.925	0.955	0.964	0.976	0.957

**Table 3:** Accuracy of the SSL-GAT and comparison methods with different training set ratios.

Training Set Proportion (%)	SSL-GAT	MLP	HSN	TL-CFD	PCGAN	PTLN
90	0.9763	0.9206	0.9662	0.955	0.9755	0.9606
80	0.9812	0.9249	0.9641	0.9566	0.9737	0.9549
70	0.9761	0.9394	0.9535	0.951	0.9642	0.9694
60	0.9734	0.9267	0.9567	0.9438	0.9568	0.9667
50	0.9809	0.9236	0.9308	0.94	0.9609	0.9536
40	0.9791	0.9398	0.9441	0.9552	0.9413	0.9498
30	0.9765	0.9243	0.9476	0.9484	0.9496	0.9543
20	0.9710	0.9266	0.9514	0.9552	0.9467	0.9466
10	0.9724	0.9227	0.9462	0.9439	0.9486	0.9427

**Table 4:** The F1-scores of the proposed method and the comparison method at different training set shares.

Training Set Proportion (%)	SSL-GAT	MLP	HSN	TL-CFD	PCGAN	PTLN
90	0.9726	0.9104	0.9463	0.9351	0.9571	0.9449
80	0.9768	0.9145	0.9454	0.9368	0.9639	0.939
70	0.9771	0.9396	0.9533	0.9474	0.9645	0.9365
60	0.9756	0.9161	0.9564	0.9533	0.9567	0.9407
50	0.9721	0.9133	0.9602	0.9567	0.9401	0.9443
40	0.9763	0.9192	0.9545	0.9656	0.9615	0.9437
30	0.9735	0.9144	0.9677	0.9682	0.9592	0.9275
20	0.9712	0.9163	0.9614	0.9618	0.966	0.9355
10	0.9734	0.9124	0.9663	0.9631	0.9683	0.9534

## Data Availability

Due to conditions set by the data providers, the data supporting the results of this study cannot be supplied to third parties.
